# Weird, Witty and Worthwhile: Exploring the Benefits of Mnemonic‐Based Medical Education

**DOI:** 10.1111/tct.70518

**Published:** 2026-09-04

**Authors:** Shazia Sarela, Malvi Shah, Emma Kelley, Nidhi Rege, Harish Bava, Rama Aubeeluck, Elena Boby, Anusha Prabhu

**Affiliations:** ^1^ University College London London UK; ^2^ King's College Hospital NHS Foundation Trust London UK

**Keywords:** medical education models, memory aids, mnemonics, student confidence, teaching strategies

## Abstract

**Background:**

Mnemonics, both self‐created and well established, are commonly used by medical students to boost their recall of knowledge. Previous studies have evaluated mnemonics' utility in medical education, but few have demonstrated positive effects on students' knowledge and confidence. Mnemonics are also more commonly shared directly between peers, as opposed to being formally taught. This study explores the impact of mnemonic‐based, near‐peer‐led teaching on medical students' knowledge and confidence. It aimed to determine whether mnemonics enhance students' ability to answer questions and boost self‐perceived confidence and future mnemonic use.

**Methods:**

Over 8 months, 23 peer‐led teaching sessions on commonly tested clinical medical topics were held for third‐year medical students, with mnemonics incorporated into all sessions. Presession and postsession SBA questions and five‐point Likert scales were used to assess change in students' knowledge and confidence, respectively. Postsession, Likert scales were used to assess students' self‐rated utility of the mnemonics taught and their likely future use of mnemonics.

**Results:**

Students demonstrated a significant improvement in SBA scores, particularly for mnemonic‐linked questions (*p* < 0.05). Confidence levels significantly increased postteaching (*p* < 0.01). Students reported a higher likelihood of future mnemonic use, and a strong correlation was found between improved confidence and increased mnemonic use (*p* < 0.001).

**Conclusion:**

Peer‐led teaching with mnemonic integration significantly improved both knowledge, student confidence and likelihood of future mnemonic use. This study highlights the potential of both mnemonic‐based and peer‐led learning strategies in medical education.

## 1 Background

Undergraduate medical education requires effective learning strategies to cope with high‐stakes written and practical exams [1, 2]. As medical students, we frequently find mnemonics, defined as ‘any device for aiding the memory’ [3], a valuable tool for managing the vast amount of information we need to retain. Mnemonics provide numerous benefits for both written and practical medical examinations, aiding the quick recall of differential diagnoses or management options and offering structured frameworks for history‐taking, examinations or procedures, particularly in high‐pressure situations [4, 5]. Different types of mnemonics include acronyms, where each letter corresponds to the first letter of a memorable word, imagery that uses visual recall, rhyme that employs memorable phrases, and connections and associations, where a phrase sounds similar to the target concept [3, 6]. Table 1 provides examples of each type of mnemonic. Students often use established mnemonics as well as self‐created ones, which may relate to their own personal contexts and experiences [3, 7, 8, 9].

**TABLE 1:** Types of mnemonics commonly used in medical education.

Mnemonic category	Example	Explanation
Acronym	ABATE the bleed (ABCDE for acute resuscitation, blood transfusion, antibiotics, terlipressin, endoscopy within 24 h)	Acute management of upper GI bleeding
Imagery	Hypocalcaemia ECG changes: lengthened QT interval à Mum and baby are far apart if there's hypocalcaemia therefore lengthened interval	Hypocalcaemia ECG changes
Rhyme	Stones, bones, thrones, abdominal groans and psychic moans	Causes of hypercalcaemia
Association	Rich Old Men only Call people	Richter's transformation of CLL to an aggressive non‐Hodgkin's lymphoma commonly occurs in older men
Connection	In DKA treat the Ketosis, in HHS treat the Hyperglycaemia	Management of diabetic emergencies

*Mnemonics provide numerous benefits for both written and practical medical examinations, aiding the quick recall of differential diagnoses or management options and offering structured frameworks for history‐taking, examinations or procedures, particularly in high‐pressure situations*.

Research into the utility of mnemonics in medical education is limited, with two studies demonstrating their effectiveness in improving differential diagnosis generation [10, 11]. Students' test scores in these studies improved following the mnemonic‐based teaching, and students found the mnemonics taught to them useful. However, only imagery‐based mnemonics yielded significant improvements in test scores.

*Research into the utility of mnemonics in medical education is limited, with two studies demonstrating their effectiveness in improving differential diagnosis generation*.

Mnemonics have also been evaluated as part of an interactive audiovisual mnemonic–based learning platform for teaching basic sciences, factual‐recall type information. Students using the audiovisual platform had significantly better immediate and delayed recall of the facts and reported significantly greater satisfaction with the learning methods compared to students taught with traditional methods [12, 13]. Mnemonic frameworks have also been proposed for recalling communication techniques in serious illness conversations and conversations about disability and teaching students about brief motivational interviewing, with many reported to be memorable and useful for students [14, 15, 16].

However, other studies have shown weaker evidence for the utility of mnemonics. A study comparing the utility of the ‘standard’ mnemonic (‘4 Hs and 4 Ts’, standing for hypotension, hypokalaemia, hypothermia, hypoglycaemia, tension pneumothorax, cardiac tamponade, thrombus and toxins) to an image‐based memory aid for recalling the causes of electromechanical disassociation in cardiac arrest demonstrated that the image‐based mnemonic was significantly more effective for immediate recall of the causes but not for delayed recall [17]. Another study using an imagery‐based mnemonic to help undergraduate students learn the Krebs cycle was unsuccessful in improving knowledge acquisition compared to the control [18].

Interestingly, in one study looking at a mnemonic to assist students in generating cardiac differential diagnoses after being provided with a case vignette, there was a decrease in the number of potentially correct noncardiac diagnoses [11]. This highlights that mnemonic‐based teaching is perhaps best in the context of aiding the learning and recall of discrete facts; using mnemonics in the context of clinical decision‐making may indeed narrow students' thinking or teach them to think mechanistically as opposed to considering the entire clinical context.

Although mnemonics have been investigated in various contexts, few studies have shown significant impacts on both test scores and student confidence. Additionally, limited research has explored the wide‐ranging applications of mnemonic‐based teaching across a range of different medical specialities or examined their impact on long‐term learning strategies.

Furthermore, most studies have investigated mnemonics as part of formal, faculty‐led teaching sessions, with limited exploration of their use in near‐peer teaching. Near‐peer teaching, defined as instruction provided by ‘a trainee one or more years senior to another’ [19], has been shown to be highly effective. Senior students can offer recent insights into examinable topics, allowing them to provide teaching focused on high‐yield concepts and topics commonly found challenging [20, 21]. Despite the recognised benefits of mnemonics and near‐peer teaching as individual strategies, there is limited research on their combined effects.

This study aimed to address this gap by investigating the impact of mnemonic‐based, near‐peer‐led teaching on medical students' knowledge and confidence. Near‐peers, having used mnemonics in their own exams, are uniquely positioned to identify concepts that benefit from memory aids and share mnemonics they found particularly effective. We hypothesised that integrating mnemonics into near‐peer teaching provides a powerful, dual approach that enhances recall, exam performance and self‐confidence.

*Near‐peers, having used mnemonics in their own exams, are uniquely positioned to identify concepts that benefit from memory aids and share mnemonics they found particularly effective*.

The primary aim of this study was to investigate whether mnemonic‐based teaching significantly affects students' performance on exam‐style questions, their confidence in examinable topics and their likelihood of using mnemonics in future learning. The secondary aim was to evaluate whether near‐peer‐led, mnemonic‐based teaching significantly improves students' ability to correctly answer exam‐style SBA questions and confidence in examinable topics.

## 2 Methods

### 2.1 Study Structure

Twenty‐three weekly 90‐min sessions were delivered over 8 months, targeted at third‐year medical students at a single UK medical school. These sessions were designed and taught by a team of 10 fourth‐year medical students, with content reviewed by qualified doctors. Sessions were held online, advertised as incorporating high‐yield curriculum content, memory aids and exam‐style questions. Attendance and participation in the teaching sessions were voluntary. Table 2 lists the topics covered. Each session incorporated at least two mnemonics linked to the content taught, categorised as one of the following types: acronyms, rhymes, associations, connections or imagery.

**TABLE 2:** Topics taught by Year 4 medical students in 23 online, voluntary teaching sessions across an 8‐month period.

Session number	Topics taught during 23 teaching sessions
1	Anaemia
2	Metabolic abnormalities
3	Electrocardiograms and chest X‐rays
4	Arterial blood gases and abdominal X‐rays
5	ABCDE structure and pharmacology
6	Acute kidney injury causes and management
7	Perioperative care and prescribing
8	Acute coronary syndrome and tachycardia
9	Type 1 & 2 diabetes
10	Heart failure and hypertension
11	LFTs and gallbladder, pancreatic and hepatic conditions
12	Bowel conditions and abdominal ABCDE situations
13	Statistics and ethics and law
14	Stomach, oesophagus and colorectal conditions
15	Pneumonia and tuberculosis
16	Fluid balance disorders
17	Thyroid & parathyroid disorders
18	Neurology emergencies and epilepsy
19	Lung cancer and pulmonary function
20	Glomerular problems and renal topics for practical exams
21	Visual field defects and headaches
22	Rheumatology antibodies and presentations
23	Haematology cancers and blood films

**FIGURE 1:**
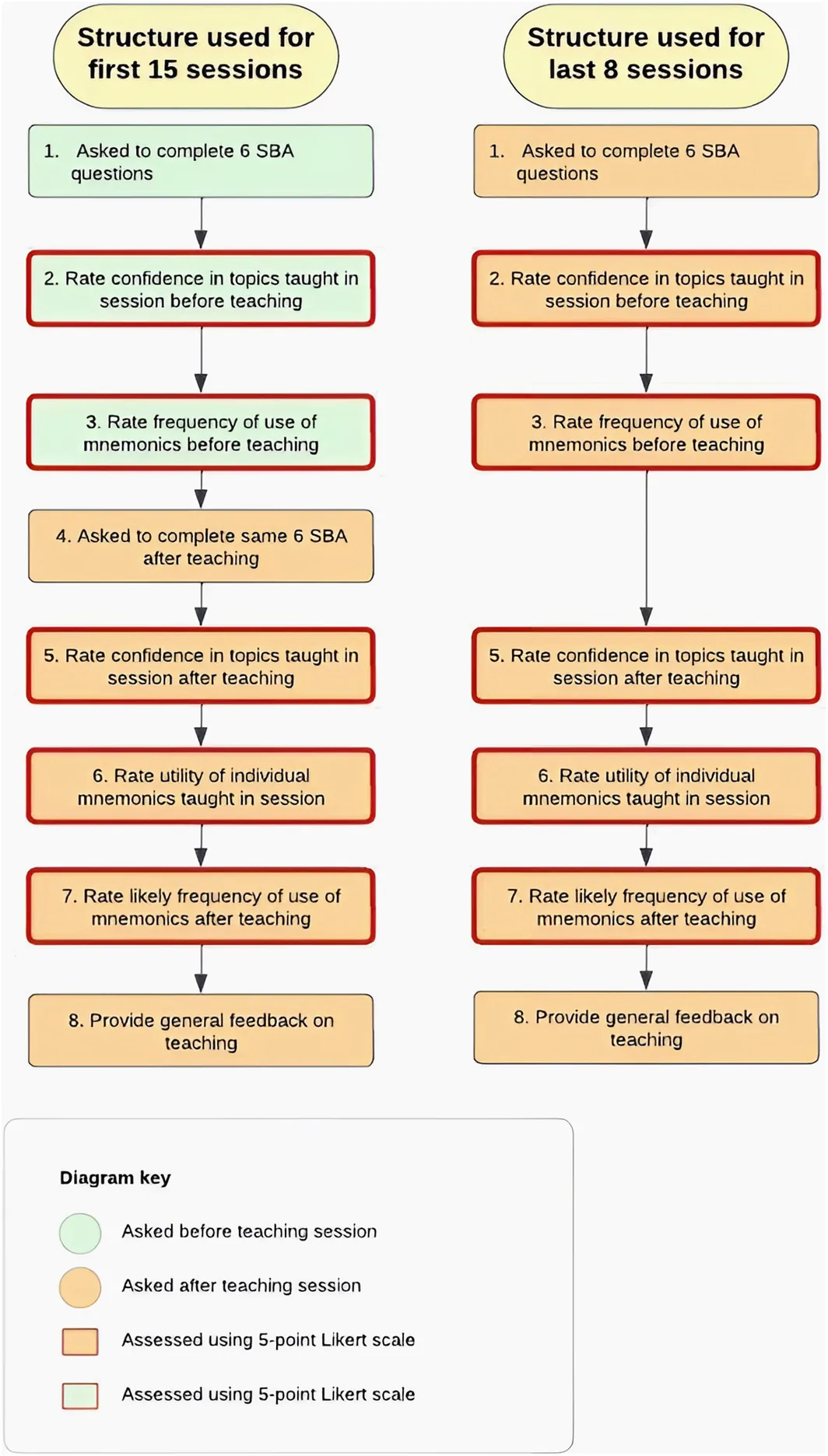
Flow chart illustrating the structure using to collect data from students across delivery of 23 teaching sessions using feedback forms.

Figure 1 outlines the structure of the feedback form used for the first 15 sessions. A 5‐\point Likert scale was used to assess students' confidence in the topics being taught before and after the session, frequency of mnemonic use before teaching; perceived utility of mnemonics taught and likelihood of future use. A score of 1 corresponded to *completely inutile*, *very poor* or *never* and 5 to *extremely utile*, *excellent* or *all the time*. SBA questions were utilised to evaluate objective performance in the content taught due to their binary nature and widespread use in UK medical school curricula and examination.

### 2.2 Mnemonic Selection

The mnemonics taught in each session were selected by the tutor delivering the tutorial. Tutors were instructed to use at least two mnemonics per session and were encouraged to include mnemonics they had previously utilised in their own learning and had found useful. The type of mnemonic or the mnemonics existing widespread use versus creation by the individual tutors was not specified to allow tutors greater freedom to include mnemonics best suited to the concept and topic they were teaching.

### 2.3 Study Design

Before the delivery of the teaching, students were asked to complete six SBA questions, as well as use a Likert scale to rate their current frequency of use of mnemonics and their current confidence in the topics being taught before the session. The SBA questions students were asked to complete included questions that could and could not be answered using the help of mnemonics taught in the lesson, with a minimum of two of each type of questions in each session. This was done to allow students' performance in nonmnemonic linked questions to serve as a ‘control’ question to be compared against those that were linked to a mnemonic.

After the session, students were asked to reanswer the same SBA questions used before the session was delivered. Additionally, they were asked to rate their confidence in the topics that had been taught after the session, their perceived utility of the mnemonics taught and their predicted future use of mnemonics after the teaching. The presession and postsession questions were matched by students completing both sections on the same form without submission of the form after the completion of the presession questions. This allowed for no identifying material to be collected to maintain anonymity.

Data were collected using Google Forms. All students attending the teaching sessions were provided with a Participation Information Leaflet, which detailed how their data would be used and their right to withdraw from the study at any time. Informed consent was obtained from all students for their anonymised data to be utilised in this study.

Based on midcycle feedback from tutors and students, the presession aspect of the feedback form was removed for the final eight sessions to expedite form completion and encourage higher response rates (Figure 1). This resulted in all parts of the survey being completed after the teaching had been delivered. Students now completed six mnemonic‐ and nonmnemonic‐based SBAs after the teaching. They were asked to retrospectively rate their confidence on the topics taught before the teaching and their frequency of mnemonics taught, followed by their current confidence after the teaching and their predicted future frequency of mnemonic usage. They rated the utility of the mnemonics taught during the teaching as requested in the preamended survey.

### 2.4 Data Analysis

Data were analysed using Python Pandas and SciPy packages. The Spearman rank correlation test was used to evaluate correlations amongst Likert scale variables and SBA scores. The Wilcoxon signed rank test assessed significant differences in paired presession and postsession data, enabling comparisons between mnemonic‐ and nonmnemonic‐linked SBA questions.

## 3 Results

A total of 147 students attended the teaching across the 23 sessions conducted, with a mean of 6.39 (range: 1–18) students attending each session.

### 3.1 SBAs to Assess Utility of Mnemonics and Overall Utility of Peer‐Provided Teaching Sessions

**FIGURE 2:**
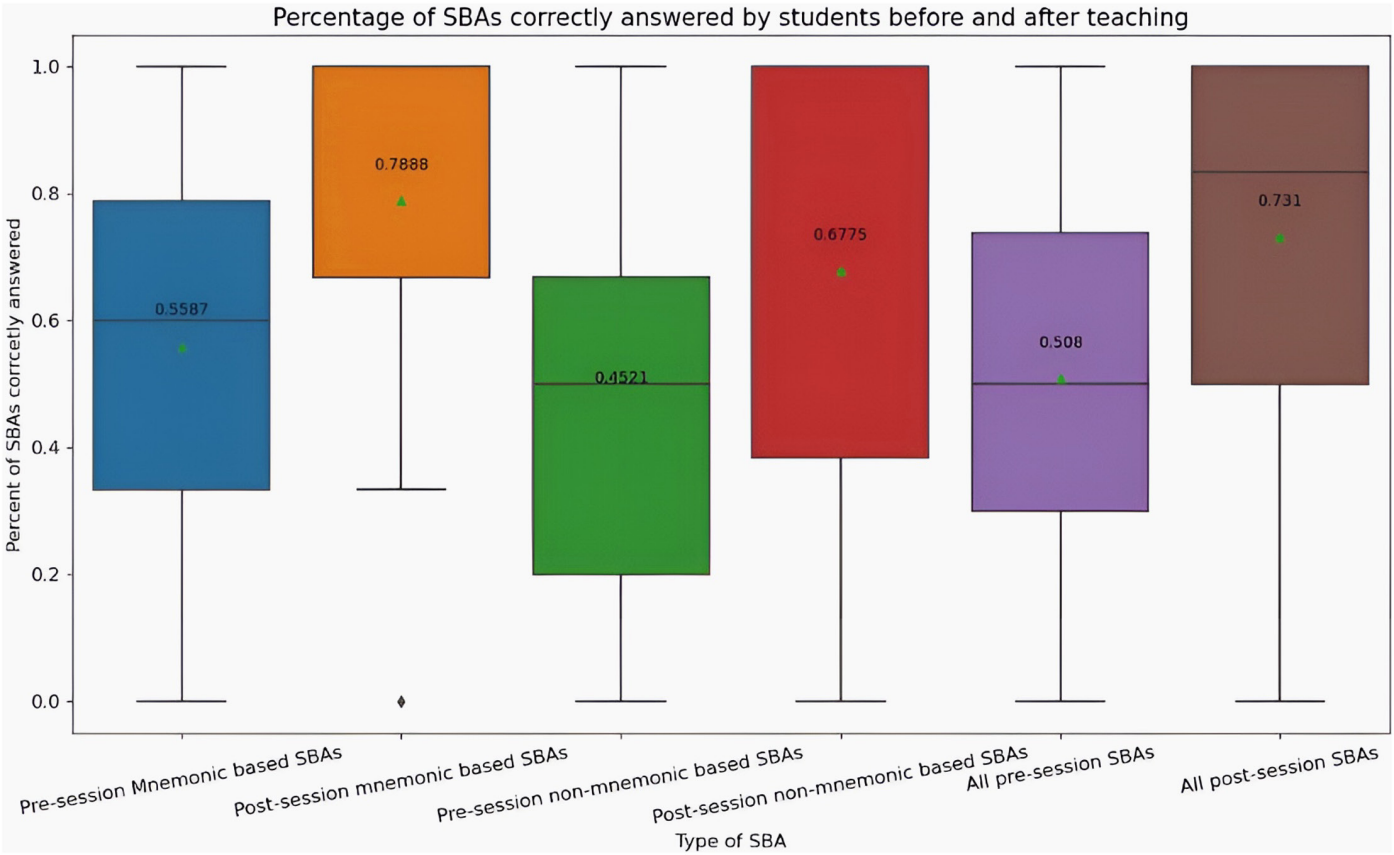
Box plot illustrating the percentage of mnemonic‐based SBAs, nonmnemonic‐based SBAs and overall SBAs correctly answered before and after the teaching session.

There was a significant improvement in the mean percentage of nonmnemonic‐ and mnemonic‐related SBAs answered correctly after teaching (mnemonic: Wilcoxon signed rank test *p* value = 5.966 × 10^−7^; nonmnemonic: Wilcoxon signed rank test *p* value = 1.630 × 10^−6^). After teaching, the mean percentage of correctly answered mnemonic‐based SBAs was significantly greater than that of nonmnemonic‐based SBAs (Wilcoxon signed rank test *p* value = 0.0055) (Figure 2).

### 3.2 Confidence in Topics Taught Before and After Delivery of Teaching Sessions

Students' mean self‐perceived confidence in the topics taught after teaching (mean: 3.9558/5) was significantly greater than their confidence before teaching (mean: 2.3321/5) (Wilcoxon signed rank test *p* value = 1.630 × 10^−6^).

### 3.3 Utility of Mnemonics Provided During Teaching Sessions

Over 65 mnemonics of a range of type and purpose were taught over the 23 sessions held. Forty‐seven percent of students rated the utility of the mnemonics in aiding their memory of the associated facts as 5/5, with 97% of all students rating the utility as greater or equal to 3/5. The mean rating of mnemonic utility across all sessions was 4.2/5.

**FIGURE 3:**
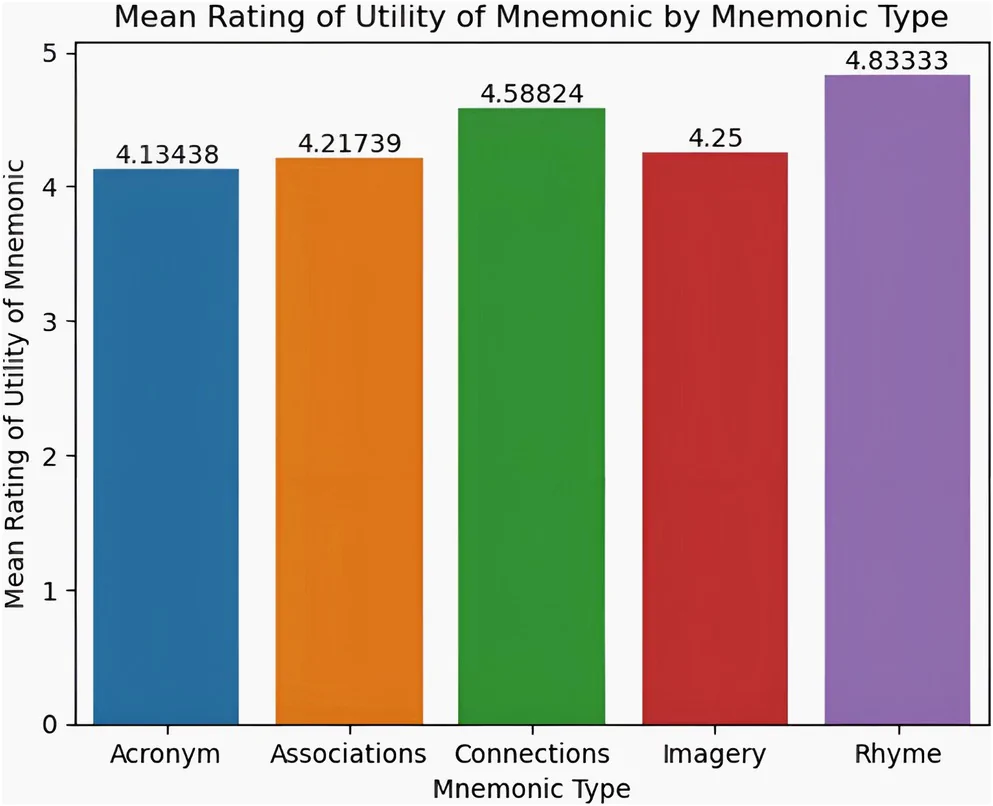
Bar charts illustrating mean students' scores for the utility of the mnemonics taught out of 5 by mnemonic type.

Acronyms were used the most frequently across all sessions (51 uses), followed by associations (six uses). Rhymes, imagery and connections were used the least frequently (three uses). Rhyme‐related questions were answered correctly 100% of the time after the teaching, followed by associations (95%). Acronyms and imagery linked questions were answered correctly 78% and 76% of the time after teaching, followed by imagery related questions answered correctly only 63% of the time. Rhymes also received the highest mean rating out of 5 for utility by the students (Figure 3).

**FIGURE 4:**
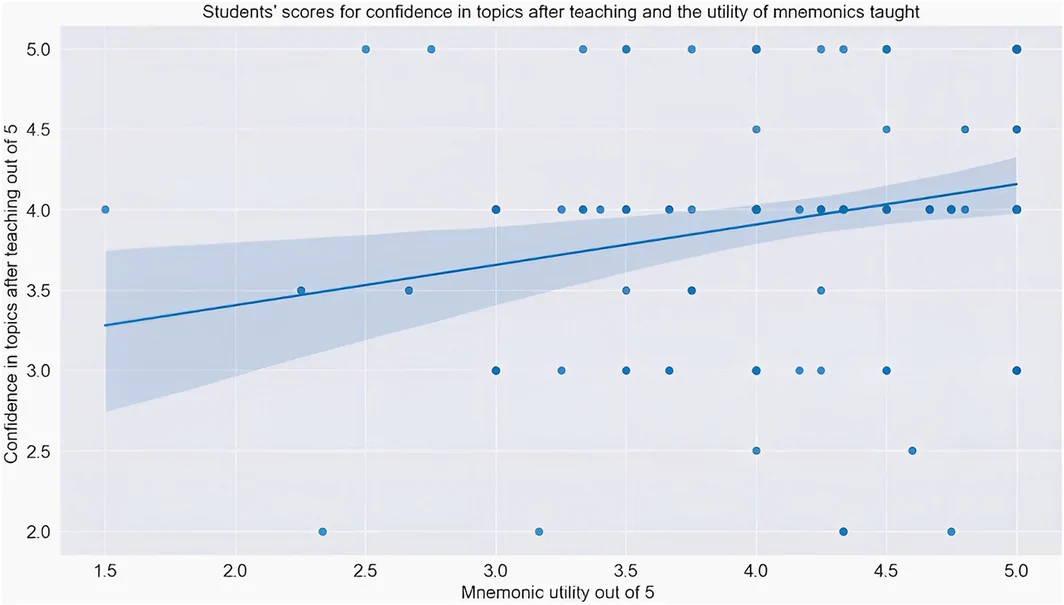
Regression plot between students' scores for their confidence in the topics taught after teaching and their scores for the utility of mnemonics provided.

Figure 4 represents the correlation between students' scores for their confidence in the topics taught after teaching and their scores for the utility of mnemonics provided. A significant positive correlation was found (Spearman rank correlation *p* value = 0.00085).

### 3.4 Frequency of Use of Mnemonics Before and After Delivery of Teaching

**FIGURE 5:**
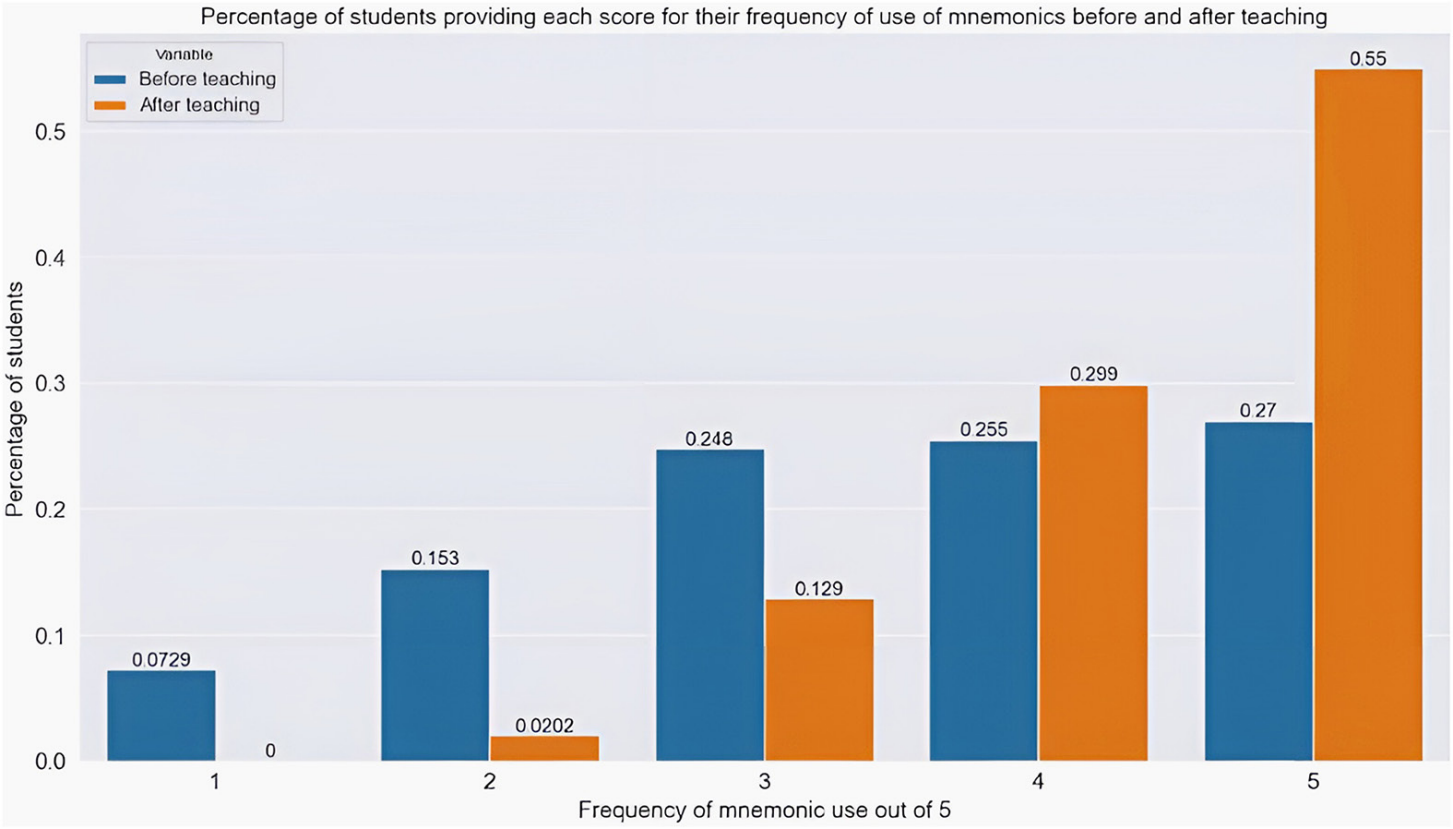
Bar chart illustrating percentage of students rating their frequency of use of mnemonics before and after teaching out of 5.

Students self‐reported likely use of mnemonics after teaching (mean: 4.381/5) was significantly greater than their use of mnemonics before attending the teaching (mean: 3.496/5) (Wilcoxon signed rank test *p* value = 1.85 × 10^−13^). Over 50% of students reported their likely future use of mnemonics after the teaching as 5/5, compared to 27% before the teaching (Figure 5).

**FIGURE 6:**
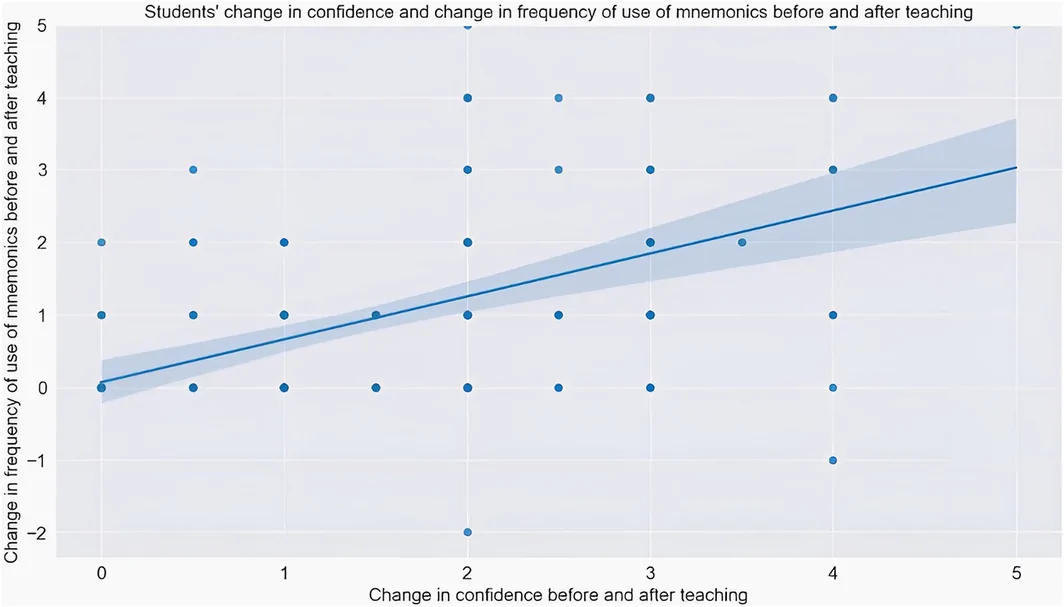
Regression plot between students change in confidence in topics taught and their predicted change in frequency of use of mnemonics before and after delivery of teaching.

Figure 6 illustrates the correlation between students' change in confidence in topics taught and their predicted change in frequency of use of mnemonics before and after delivery of teaching. A significant positive correlation was found (Spearman rank correlation *p* value = 1.157 × 10^−9^).

**FIGURE 7:**
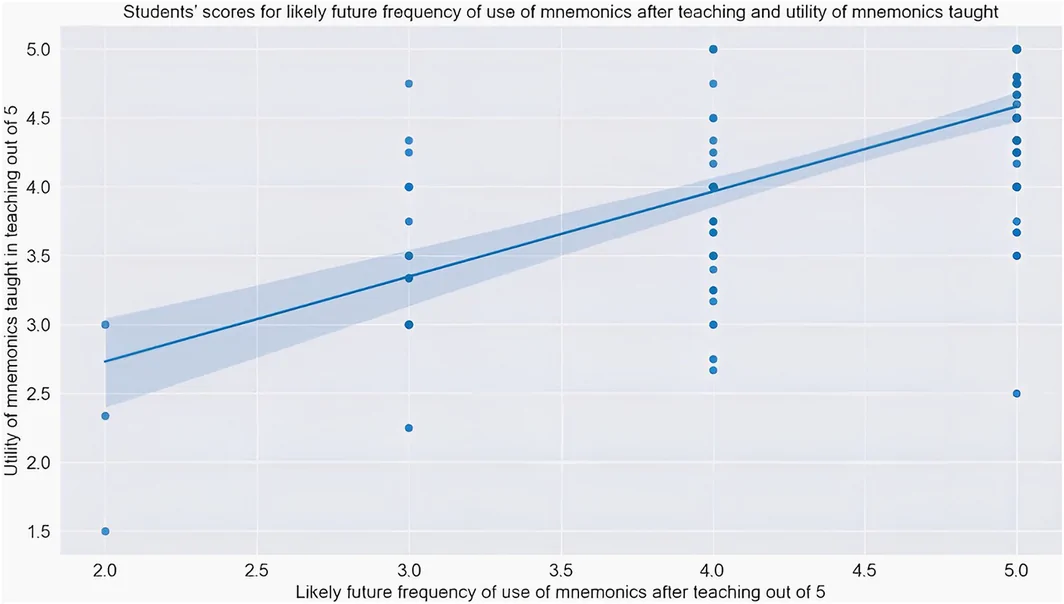
Regression plot between students' scores for likely future frequency of use of mnemonics after teaching and their scores for usefulness of mnemonics taught.

Figure 7 illustrates the correlation between students' scores for their likely future frequency of use of mnemonics after teaching and their scores for the usefulness of mnemonics taught. A significant positive correlation was found (Spearman rank correlation *p* value = 1.278 × 10^−17^) (Figure 7).

## 4 Discussion

In this study, the use of mnemonics significantly enhanced students' performance in SBA questions. Students performed better in SBA questions that could be answered with the help of mnemonics compared to those that were not assisted by mnemonics. This suggests that the ability to recall facts about a medical topic using mnemonics may improve students' performance in assessment. This finding aligns with previous studies demonstrating the positive impact of mnemonics on students' knowledge, including differential diagnosis generation and multiple‐choice test scores [10, 11, 12, 13, 22].

Our sessions aimed to teach clinical concepts from first principles, before incorporating mnemonics as a memory aid. Mnemonics were not taught in isolation but within sessions on a particular topic. By contrast, studies investigating mnemonics in isolation did not find a significant impact on students' question‐answering performance [17, 18]. This suggests that mnemonics may be most effective when introduced after students have developed sufficient baseline knowledge.

In our sessions, mnemonics were used to teach students discrete, hard to remember facts, judged by tutors to be important for exams. Other studies that used mnemonics to teach complicated concepts, such as the Krebs' cycle or the action of the extraocular muscles, showed less benefit to students' assessment scores [18, 23] This suggests that mnemonics may be less suitable for concepts where the mnemonic itself is too complex to be recalled.

In addition to performance improvements, students' self‐rated confidence levels increased significantly, which correlated with a greater projected increase in frequency of use of mnemonics after teaching. Previous studies have also reported increases in student confidence and future mnemonic use, though the correlation between them was not assessed [10, 22].

*In addition to performance improvements, students' self‐rated confidence levels increased significantly, which correlated with a greater projected increase in frequency of use of mnemonics after teaching*.

Students also reported finding the mnemonics taught in our sessions very useful. Learners have found a wide range of other mnemonics useful [10, 12, 13, 22]. Previous studies have shown that students find mnemonic‐based teaching more enjoyable than standard teaching [22], suggesting the benefits of mnemonics in topics that students normally find to be uninspiring or tedious to learn.

The mnemonics utilised in this study were selected at the discretion of the individual tutors without any prior restriction on type or source, allowing tutors to select those best suited to the topics taught. Other studies demonstrating improvements in knowledge or confidence also utilised similarly selected mnemonics [10, 11, 22]. By contrast, a study where students created mnemonics themselves and shared them with their peers found no improvement in performance or confidence compared to controls [24]. This suggests that established mnemonics, selected by senior students with understanding of their utility, are more helpful than those devised specifically for teaching. Future studies can select mnemonics for evaluation through greater peer discussion and could compare the exam performance and perceived utility of established mnemonics with those created specifically for teaching.

The significant improvement in students' confidence and overall scores in SBAs also supports our secondary aim of demonstrating the benefits of peer‐led teaching alongside mnemonic‐based learning. Senior students, having recently completed the same exams and curriculum, are ideally placed to assist junior students. Near‐peer teaching also shows benefits teachers by providing them with experience, confidence and leadership skills [20, 21]. The impact on tutors and the wider medical school community was not specifically evaluated in this study though tutors anecdotally reported positive impacts on their personal development and students continued to reach out to tutors after the sessions.

Of the five types of mnemonics provided to students, rhymes were rated the most useful. Furthermore, SBAs that could be answered with rhyme‐type mnemonics were the most likely to be correctly answered. Rhymes may offer superior memorability due to their rhythmic and melodic nature, compared to acronyms, which require additional cognitive effort to unpack. However, creating ‘rhymes’ is challenging, resulting in their use only three times across the study. In contrast, acronyms were utilised over 50 times due to their frequent use to recall common causes of diseases, frameworks for treatment or lists of drug side effects. Future studies could standardise the frequency of different mnemonic types to better assess their relative utility.

Our findings revealed minimal variation in confidence, SBA scores and utility of mnemonics across all sessions, suggesting our findings may be applicable across the range of topics taught. However, large variations in the number of attendees between sessions, ranging from 1 to 18, prevented topic‐ and speciality‐specific analysis. A study with a larger, consistent group of participants at each session would allow for this to be addressed. Our sessions mainly focused on diagnosis, investigations and management of common conditions. Future sessions evaluating mnemonics on communication skills and decision making would be important, as many mnemonics have been proposed in these areas but not rigorously evaluated [25, 26, 27].

A significant limitation of our study was the loss of presession data for the last eight sessions following amendments to the feedback form to maximise completion. Students were required to retrospectively rate their presession confidence and frequency of mnemonic use, potentially introducing bias. The lack of presession SBAs prevented assessment of changes in SBA scores for these sessions. Future studies should incorporate presession and postsession assessments into formal evaluation.

Another limitation was the inability to link data from students who attended multiple sessions because identifying data were not collected. This prevented evaluation of whether greater attendance improved SBA scores and usage of mnemonics. Future could assign participants unique, randomly generated identifiers to allow anonymous linkage across sessions. Additionally, mnemonic utility was assessed immediately after teaching, evaluating short‐term recall. Longer term follow‐up would allow assessment of effects on recall, performance, confidence and understanding. Previous studies assessing longer term retention found that knowledge reduced over time, suggesting that repeated refreshers' or students' take‐home resources may be beneficial [12, 13].

Another limitation was the inability to definitively attribute differences between mnemonic and nonmnemonic SBA scores to mnemonic use rather than differences in question difficulty. Although both groups showed significant improvement following the intervention, postsession mnemonic scores were significantly higher than nonmnemonic scores, whereas baseline mnemonic scores were also higher. This may reflect variation in question difficulty, prior familiarity with content, mnemonic strategies or chance. Although the relatively large dataset (six questions per student across 23 sessions, involving over 140 participants) was intended to mitigate this issue, residual confounding may remain. Future studies could use independent, blinded raters to assess and standardise the perceived difficulty of mnemonic and nonmnemonic questions, improving comparability and causal inference.

Finally, all variables were assessed using SBA questions and 5‐point Likert scales. Future studies incorporating qualitative methods, such as structured interviews and free‐text questions, could provide greater insight into students' perceptions of mnemonic‐based peer‐led teaching.

## 5 Conclusion

In conclusion, this is the first study to formally evaluate the impact of mnemonic‐based, peer‐led teaching on medical undergraduate students' ability to correctly answer exam‐style SBA questions, enhance confidence and promote long‐term mnemonic use. The findings underscore the significant benefits of peer‐led teaching and the use of mnemonics in medical education. The marked improvement in SBA scores and student confidence following the teaching sessions demonstrates the effectiveness of senior students in facilitating mnemonic‐based teaching for their junior peers. Mnemonics, when carefully selected and integrated into teaching, proved to be highly valuable in improving both short‐term recall and student engagement with examinable topics. Finally, the strong correlation between mnemonic use and confidence establishes the critical role for mnemonic‐based, peer‐led teaching in enhancing medical education, highlighting the need to incorporate these strategies into curricula to support deeper learning and sustained knowledge retention.

*Mnemonics, when carefully selected and integrated into teaching, proved to be highly valuable in improving both short‐term recall and student engagement with examinable topics*.

## Author Contributions

**Shazia Sarela:** conceptualization, investigation, writing – original draft, writing – review and editing, methodology, formal analysis. **Emma Kelley:** writing – review and editing, supervision. **Harish Bava:** writing – original draft. **Elena Boby:** writing – original draft. **Anusha Prabhu:** writing – original draft. **Rama Aubeeluck:** writing – original draft. **Nidhi Rege:** writing – original draft. **Malvi Shah:** conceptualization, investigation, writing – original draft, writing – review and editing, methodology, formal analysis.

## Funding

The authors have nothing to report.

## Ethics Statement

The need for ethics approval for this study was waived by the University College London Research Ethics Committee (UCL REC), as it collected completely anonymous, nonsensitive data from students. This study adhered to the Declaration of Helsinki. Informed consent to participate in this study was obtained from all participants.

## Consent

The authors have nothing to report.

## Conflicts of Interest

The authors declare no conflicts of interest.

## Data Availability

The data that support the findings of this study are available from the corresponding author upon reasonable request.
